# Perineal and right femoral hydatid cyst in a female with regional paresthesia: a rare case report

**DOI:** 10.1186/s12893-022-01516-z

**Published:** 2022-02-23

**Authors:** Mojtaba Ahmady-Nezhad, Ramin Rezainasab, Armin Khavandegar, Samaneh Rashidi, Sanaz Mohammad-Zadeh

**Affiliations:** 1 Surgery Department, Madani Hospital, Alborz University of Medical Sciences, Karaj, Iran; 2 Student Research Committee, School of Medicine, Alborz University of Medical Sciences, Karaj, Iran; 3 Pathology Department, Madani Hospital, Alborz University of Medical Sciences, Karaj, Iran; 4 Department of Clinical Research Development, Madani Hospital, Alborz University of Medical Sciences, Karaj, Iran

**Keywords:** Hydatid cyst, Inguinal hydatid cyst, *Echinococcus granulosus*, Femoral hydatid cyst, Multi-lobular cystic mass, Spinal paresthesia

## Abstract

**Background:**

Hydatid cyst is a zoonotic disease caused by the parasite *Echinococcus granulosus*. The tapeworm larvae can create cyst in different areas of the body, especially the liver and lungs; however, the formation of the cyst in the perineal and femoral regions are very rare. The unusual location of the cyst can help us with the differential diagnosis of soft tissue mass(es) in this location, especially in endemic areas. Diagnosis of this disease is crucial because if the cysts are ruptured during surgery, the fluid inside can cause anaphylactic shock.

**Case presentation:**

Our case is a 55-year-old woman with the chief complaint of a painful mass in the right thigh and perineal area with progressed pain and paresthesia to the right thigh and shin. The patient had no history of fever, abdominal pain, digestive dysfunctions, or chest pain. The vital signs were normal, and there was no family history. Physical examination showed that the skin over the mass had no discoloration, and the size was around 5.7 cm long. The result of the ultrasonography examination showed a cystic mass with suspicion toward the femoral hernia. After a CT scan, the result of secondary workups was a multi-lobular cystic mass with no connection to the abdominal region, which suggested a hydatid cyst. The patient underwent spinal anesthesia and surgery, a cystic mass with ecto- and endocyst, with clinical similarity to a hydatid cyst, was removed with wide margins, and the cyst wall was kept intact. In the next step, the specimen was sent for histological examination that confirmed cystic hydatidosis. The Post-surgical Abdominal and thoracic Ultrasonography screening were used to exclude relapse, and medical therapy was given for 3–6 months. An 18-months follow-up demonstrated no reoccurrence and no newly formed cyst.

**Conclusions:**

Although rare, femoral hydatid cyst can occur in some cases, especially in endemic areas. We highly recommend our colleagues consider ruling out cystic hydatidosis in any patients complaining of regional mass(es), mostly painless, presenting with adjacent tissue compression with or without manifestation of an allergic reaction.

## Background

Hydatid cyst is a chronic parasitic disease caused by four species of a famous family of parasitic tapeworms called echinococcus, most commonly, *Echinococcus granulosus* [1]. Cystic hydatidosis has a worldwide distribution and is endemic in areas with low sanitation and rural areas with poor housing conditions where humans live in close contact with cattle and canines, especially dogs [2–4]. This parasite is transmitted through contamination of food and water with its eggs found in definitive hosts like dogs’, wolves’, and foxes’ feces [5].

In some rare cases, the cysts are implanted in the areas including the inguinal canal, perineal region, spleen, skeleton, brain, kidney, peritoneum, thyroid, and parathyroid. In other words, hydatid cyst can be found “*from head to toe*” [6, 7].

In this report, we represent a rare case of perineal and femoral hydatid cyst. The unusual location of the cyst can help us with the differential diagnosis of soft tissue masses in this location, especially in endemic areas where unusual hydatid cyst formation is observed. Diagnosis of a hydatid cyst is crucial because if the cysts are ruptured during operation, the fluid inside the cyst can cause anaphylactic shock inside the body. Therefore, preoperative diagnosis of the hydatid cyst is necessary for proper treatment.

## Case presentation

A 55-year-old female was admitted to the hospital’s emergency department with the chief complaint of a painful mass located in the right thigh and perineal area and with progressed pain and paresthesia to the right thigh and right shin. The patient seemed ill but not toxic. No history of fever, abdominal pain, digestive dysfunctions, chest pain, cough, hemoptysis, urticarial were found. The vital signs were in a normal range, and there was no family history.

**Fig. 1:**
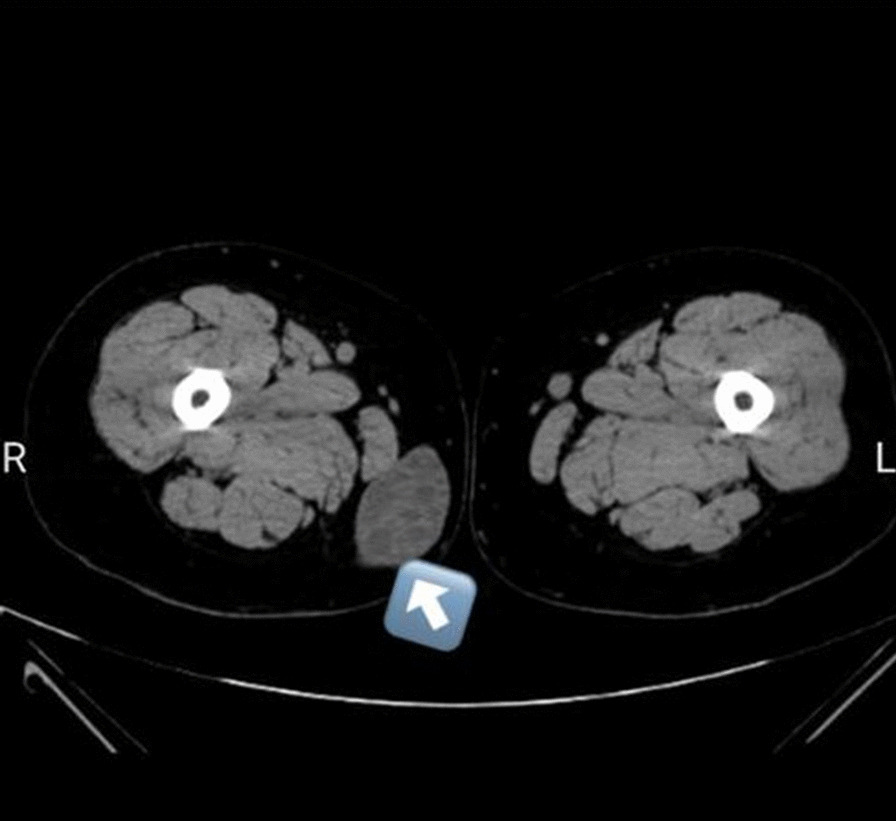
Computed tomography scan finding correlated with hydatid cysts. Multi-lobular cystic mass with no connection to the abdominal region is also observed

Physical examination showed that the skin over the mass had no discoloration, and the size of the mass was around 5.7 cm long. The blood tests were normal, and the lab results showed no eosinophilia. The result of the ultrasonography examination was a cystic mass with suspicion toward femoral hernia. After a CT scan, the result of secondary workups was a multi-lobular cystic mass with no connection to the abdominal region (Fig. 1), which suggested a hydatid cyst.

The patient underwent surgery under spinal anesthesia in the next step, and the right thigh was opened up. A cystic mass with ecto- and endocyst with clinical similarity to a hydatid cyst was removed with wide margins and without penetration of the cyst wall. The removed specimen was sent for histological examination with the first impression of cystic hydatidosis.

**Fig. 2:**
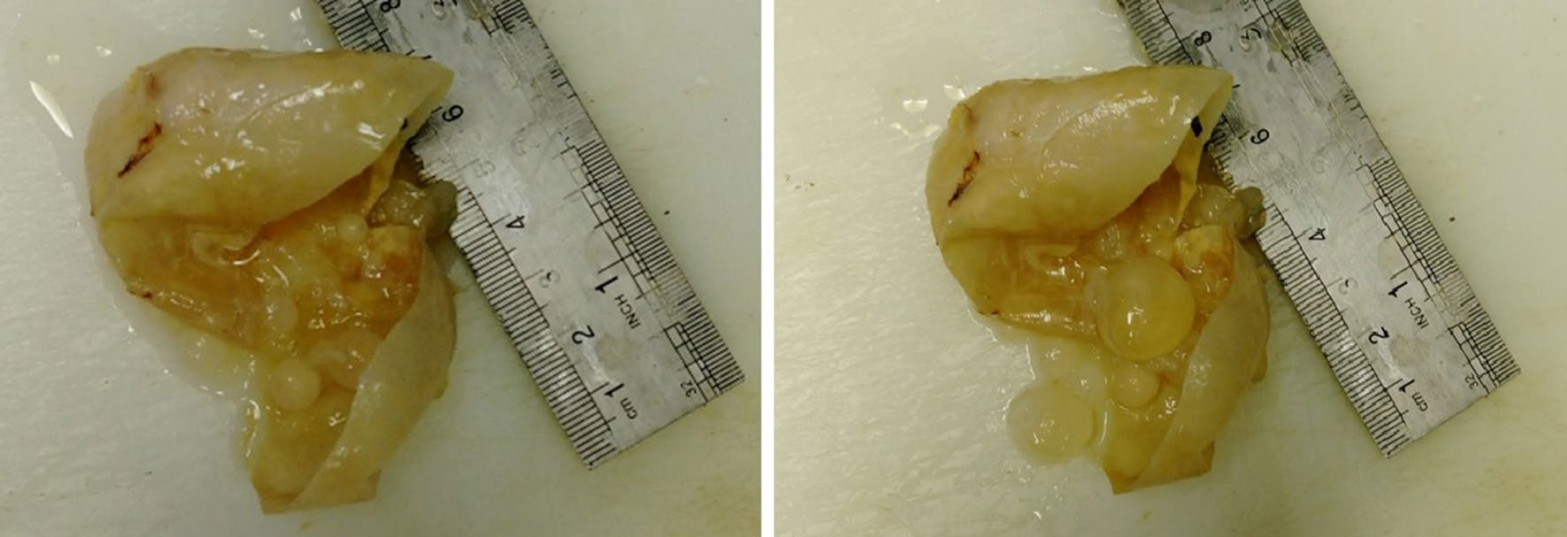
Macroscopic findings of extracted hydatid cyst. Creamy-whitish colored cyst M = 7 * 5 * 3 centimeter filled by multiple variable-sized creamy cysts

**Fig. 3:**
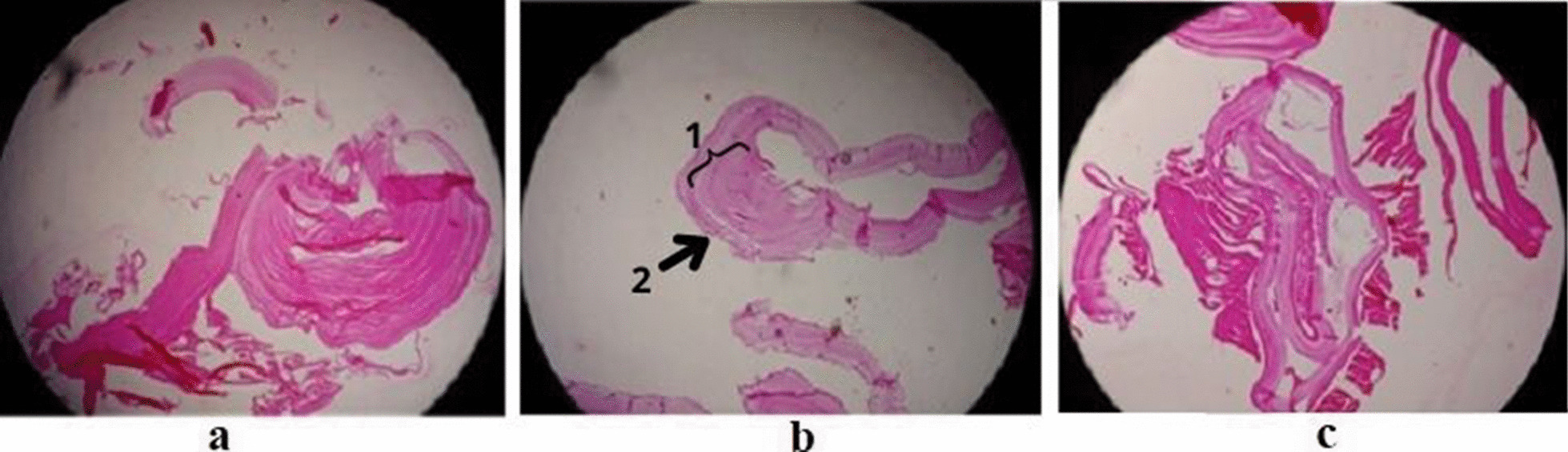
Microscopic findings of hydatid cysts. The cyst consists of three layers; outermost fibrous pericyst layer, **B** (1) middle laminated ectocyst layer, and **B** (2) the inner hyaline and acellular endocyst as the germinative layer encompasses daughter cysts and brood capsules with scolices

Received specimen in formalin consist of a creamy-whitish colored cyst M = 7 * 5 * 3 cm filled by multiple variable-sized creamy cysts (Fig. 2). The cyst consists of three layers; the outermost fibrous pericyst layer, the middle laminated ectocyst layer, and the inner hyaline and acellular endocyst as the germinative layer, which encompasses daughter cysts and brood capsules with scolices. There may be granulomatous palisading reaction and pseudocyst formation as seen in cutaneous lesions (Fig. 3).

Following the surgical removal of the cyst, the patient underwent recovery. Besides, Post-surgical Abdominal and thoracic Ultrasonography screening were used to exclude recurrence. Albendazole 400 mg PO BID was prescribed for 3–6 months. An 18-months follow-up demonstrated no reoccurrence or any other site of cystic hydatidosis.

After a year of routine follow-up, the patient did not demonstrate any signs of recurrence of the adverse effect of surgery, and she was entirely well.

## Discussion

Hydatid cyst is a disease that can cause Public health challenges, especially in endemic areas that consist of tepid climate countries, including the Mediterranean region, Central Asia, South America, Africa, and China [8]. Also, the South American countries, Iceland, Australia, New Zealand, and sub-Saharan countries are intensive endemic areas for this disease [9]. This disease usually affects the liver in 68.8–80% of cases and lungs 10–22.4% of patients [5, 6]. (especially the right lobe of the lung compared to its left lobe because of its inclination [10, 11]). However, in endemic areas, this disease can implant in rare parts of the body, including the inguinal canal, perineal region, spleen, skeleton, brain, and kidney. In other words, hydatid cyst can be found “*from head to toe*” [6, 7]. This disease has a fecal-oral route of transmission, and it enters the human body through ingestion of contaminated food and water by feces of canines containing parasites’ eggs or ova. After ingestion, these eggs turn to larva and enter the bloodstream by penetrating the intestinal wall, and from there, they go to different locations of a human’s body to create cyst(s). Also, the annual growth rate of hydatid cyst is estimated at around one three centimeters in diameter [12].

In our case, the hydatid cyst was present in the perineal and right femoral region, which is a rare area for the formation of this cyst. The exact mechanism for explaining the implantation of *Echinococcus granulosus* larva in this location is still a mystery; however, the primary distribution of this parasite by blood is probably the most reasonable explanation [11, 13]. The cysts usually manifest as slow-growing mass(es) of soft tissue, and in some cases, inflammatory symptoms and fistulization might accompany the symptoms of the cyst. The severity of symptoms varies between patients. Some cysts, almost in 75% of cases, collapse and might disappear or calcify, which are usually indolent, whereas in other cases, cysts might get more extensive and affect healthy organs and tissues by displacing or compressing them [14]. The differential diagnosis of hydatid cysts in this area is an inguinal hernia, lymphangitis, hydrocele, other types of cysts [13, 15].

In femoral or perineal hydatidosis, the preoperative diagnosis of the disease is crucial because the cyst contains a fluid inside it, which has specific antigens and, if ruptured during operation, can cause a type 3 hypersensitivity reaction. This reaction can vary from benign symptoms such as benign urticaria, episodic chills, or fever to more severe and fatal symptoms, such as edema and ultimately anaphylactic shock [16, 17]. Therefore, this disease must be considered during differential diagnosis, especially in areas with high prevalence, to avoid this phenomenon.

The diagnosis of a hydatid cyst is mainly constructed around the patient’s history, clinical findings, and examinations. The examinations consist of Serology test, Ultrasonography, with the best efficacy for detecting cysts as small as 1 cm [18, 19], CT scan, with a sensitivity of 100% [20], and Histopathological study of biopsy [21, 22]; however, performing biopsy can be dangerous, due to risk of the cyst rupture. Also, some parts of the cyst can get dislocated and migrate to other areas of the body and create secondary cysts [7]. Serology tests can help diagnose the disease and follow-up of the hydatidosis reoccurrence or new cyst formation in other parts of the body [22, 23]. Preoperative diagnosis of cystic hydatidosis, especially in non-endemic areas and rare sites of cyst formation, is complex and can sometimes be misdiagnosed with an abscess [24, 25] and dermoid cyst [9]. Therefore, diagnosis in such cases is usually made possible by exploring the site of mass or lesion intraoperatively [9]. Although the diagnosis of hydatid cyst in rare locations can be suspected during surgery, its definitive diagnosis is only achieved by pathological examination [11].

The treatment options for this disease consist of operative and non-operative methods. In this study, we prescribed albendazole alongside surgical procedures, pre-and post-operatively. The best way to treat hydatid cyst is Surgery, and medication alone is insufficient [26, 27]. Complete removal of the cystic mass alongside medical therapy is the best option for treating solitary cysts.

To detect recurrence or secondary formation in other parts of the body at an early stage, follow-up and patients monitoring by using radiological and serologic applications are necessary. The protocol for monitoring patients consists of a complete physical examination in 3, 6, 12, 18 months and, after that, annually for 10 years. In addition, serologic test, chest X-ray, hepatic sonography, and sonography of the area where the primary mass(es) were found is necessary [15].

Although rare, femoral hydatid cyst can occur in some cases, especially in endemic areas. Variations of patients’ manifestation depend on adjacent compressed tissue. We highly recommend our colleagues consider ruling out cystic hydatidosis in any patients complaining of regional mass(es), mostly painless, presenting with adjacent tissue compression with or without manifestation of an allergic reaction.

## Data Availability

All essential data have been included in this manuscript.

## Abbreviations

cm: Centimeter
CT: Computed tomography
M: Measure
mg: Milligrams
PO: Per os; by mouth
BID: Bis in die; twice a day
